# Empirically developed dietary inflammatory potential (EDIP) in patients candidate for coronary artery bypass grafting surgery (CABG): Association with metabolic parameters, dietary antioxidant quality score and dietary phytochemical index

**DOI:** 10.1371/journal.pone.0208711

**Published:** 2018-12-17

**Authors:** Mahdieh Abbasalizad Farhangi, Mahdi Najafi

**Affiliations:** 1 Nutrition Research Center, Tabriz University of Medical Sciences, Tabriz, Iran; 2 Department of Research, Tehran Heart Center, Tehran University of Medical Sciences, Tehran, Iran; 3 Cardiac Outcome Research and Education (CORE), Universal Scientific Education and Research Network (USERN), Tehran, Iran; University of Bologna, ITALY

## Abstract

**Aims:**

It has been suggested that empirically developed dietary inflammatory potential (EDIP) is a diagnostic tool for assessment of inflammatory potential of diet in prediction of risk factors related to chronic disease. In the current work, we examined the association between EDIP with cardio-metabolic risk factors, dietary antioxidant quality score (DAQs), dietary phytochemical index (DPI) and Mediterranean dietary quality index (MEDQI) in patients candidate for CABG.

**Materials and methods:**

In the current cross-sectional study, the data obtained from Tehran- Heart Center-Coronary Outcome Measurement (THC-COM) study from 454 patients candidate for the CABG were used. Laboratory measurements including hemoglobin (Hb)-A1C, serum lipids, creatinine, blood urea nitrogen (BUN), hematocrit, Lp(a), telomerase activity, serum vitamin D and c-reactive protein (CRP) were measured with commercial laboratory methods. Dietary indices were measured according to the data obtained from with semi-quantitative FFQ.

**Results:**

In the current work, patients in top quintile of EDIP had higher BMI and higher prevalence of hyperlipidemia compared with patients in lowest quintile (BMI: 28.08 ±3.68 vs 26.69 ± 3.67 and hyperlipidemia: 77.8 vs 65.5%; P < 0.05). Moreover, lower EDIP scores were accompanied with higher vitamin E (0.48 vs 0.4) and total dietary antioxidant scores (4.40 VS 4.28), higher dietary phytochemical scores (69.79 vs 58.29) and lower dietary Mediterranean quality scores (P < 0.05 and P < 0.01 respectively). In male patients, being at highest quintiles of EDIP make patients 2–5% more likely to have higher concentrations of serum cholesterol, BUN and Lp (a), and 6 to 8 times more likely to have higher creatinine and 66% more likely to have lower albumin concentrations compared with male patients in lowest quintiles. In female patients, lower HCT, higher creatinine, higher CRP concentrations and higher telomerase activity were also achieved by higher EDIP scores (P < 0.05).

**Conclusion:**

According to our finding, EDIP was associated with obesity, high prevalence of dyslipidemia and cardio-metabolic risk factors. Moreover, EDIP was in an inverse association with dietary antioxidant quality score and dietary phytochemical index. Therefore, EDIP could be assumed as a precise tool for estimating the CVD related risk factors among patients candidate for CABG.

## Introduction

According to the reports of world health organization (WHO) cardiovascular diseases (CVDs) are the number one cause of mortality worldwide and an estimated 17.7 million people died from CVDs in 2015, representing 31% of all global deaths. Of these deaths, an estimated 7.4 million were due to coronary heart disease and 6.7 million were due to stroke [1]. The treatment costs of CVD imposed on individual and society are substantial; since it involves majority of young working and productive population especially in Iran and low income countries, its heavy financial burden in these countries is alarming [2]. In Iran, coronary artery disease (CAD) is a major cause of mortality and morbidity and accounts for nearly 50% of all deaths and 79% of deaths due to chronic disease per year [3]. As estimated, the burden of cardiovascular disease in Iran will increase steeply over 2005 to 2025, mainly because of the major epidemiologic and demographic transitions and increase in aging population [4]. These alarming points highlight the need for more attention to deal with the impact of CVD in the following decades in Iran.

Primary prevention of cardiovascular disease is an important priority for developers of health policy against cardiovascular disease; healthy dietary habits, smoking cessation, weight management, regular physical activity and stress management all are most important strategies to reduce the risk of CVD later in life [5]. Diet, undoubtedly, is one of the most important factors affecting cardiovascular health and numerous healthy dietary guidelines have been developed like dietary approach to stop hypertension (DASH) recommending diets high in fruits, vegetables, polyunsaturated fatty acids and low in fat and sugar, European society of cardiology (ESC) and National Institute for Health and Clinical Excellence (NICE) recommendations about reducing saturated fat and alcohol and increase in fiber and fish intake as a hallmark of Mediterranean dietary pattern [6–8]. However, it is important to study the effect of whole diet and food groups rather than single nutrients or foods to achieve a reliable perspective of the diet-disease relationships. Recently, dietary indices and patterns have been developed and attracted much attention because of capturing multiple dietary factors and providing a comprehensive assessment of diet, accounting for the complex interactions between nutrients and foods. Because of the pro-inflammatory and anti-inflammatory roles of nutrients, the inflammatory potential of diet could be associated with numerous inflammation-associated diseases including CVD, diabetes, obesity and so on. Therefore, previous literature review based-cross sectional studies have developed dietary inflammatory indices to reveal the inflammatory potential of diet [9, 10]. Numerous reports are available about the association between dietary inflammatory index and several chronic diseases [11, 12]. However, several inconsistencies about the association between DII and chronic disease are also present [13, 14]. Recently, empirically developed dietary inflammatory potential (EDIP) has been developed in a US-based prospective cohort according to the inflammatory potential of food groups and its validity has been evaluated [10]. Recent studies have reported that EDIP has a higher ability to predict the plasma concentrations of circulating inflammatory parameters compared with DII [15].

Because of the potential role of inflammation in the pathogenesis of cardiovascular disease, and the clear association between dietary parameters with the risk of CVD, in the current study, we aimed to: (a) evaluate the role of EDIP in the prediction of cardiovascular metabolic risk factors and (b) to evaluate the association of EDIP with several other dietary indices including dietary antioxidant quality score (DAQs), dietary phytochemical index (DPI) and Mediterranean dietary quality index (MEDQI) in patients candidate for CABG.

## Materials and methods

### Subjects

The reports of the current study are a part of the Tehran- Heart Center-Coronary Outcome Measurement (THC-COM) study. Four hundred fifty four patients candidate for the CABG aged between 35 to 84 years old admitted to the cardiothoracic ward for CABG surgery at a large Heart Center in Tehran had been participated in the current study. Demographic, anthropometric and biochemical assessments were performed in participants after obtaining written informed consent. The study protocol was also approved by the ethics committee of Tehran University of Medical Sciences and Ethics committee of Tabriz University of Medical Sciences. Anthropometric assessments included weight and height which was measured by standard methods and body mass index (BMI) was also calculated. Biochemical assessments were also previously described unless about serum telomerase and vitamin D assessments which were measured by ELISA method (MyBiosource, USA).

### Measurement of empirically developed dietary inflammatory potential (EDIP), dietary antioxidant quality score (DAQ), dietary phytochemical index (DPI) and Mediterranean dietary quality index (Med-DQI)

Dietary intake was assessed using 138-item semi-quantitative food frequency questionnaire (FFQ) consisting of a list of foods with standard serving sizes commonly consumed by Iranians and was validated and adopted for use in Iran [16]. For measurement of the inflammatory potential of diet, EDIP was developed based on food group intakes. Dietary intakes were assigned into the fifteen food groups, including coffee, tea, dark yellow and leafy green vegetables, tomatoes, other vegetables, processed meat, red meat, organ meat, other fish, snacks, fruit juice, pizza, refined grains and high-energy beverages. Wine, beer and low-energy beverages were not used to construct EDIP score because their consumption is not usual in our population. Mean daily intake of each food group was determined by defined serving sizes and then weighted by the proposed regression coefficients. The weighted intake of food groups was summed to construct EDIP and then rescaled by dividing by 1000 to decrease the magnitude of the score and simplify the interpretation [10].

Total dietary antioxidant quality score (DAQ) was calculated according to a method first described by Rivas et al [17, 18]. For the calculating the score, the intake of several certain antioxidants including zinc, selenium, vitamin A, vitamin E and vitamin C were assessed separately by assigning a score of 0 or 1. This scoring was based on the comparison of nutrient intake with the dairy recommended intake of nutrients (RDA). When the intake was below 2/3 of the RDA, 0 score was signed. While in the dietary intake of nutrient higher than 2/3 RDA, the assigned score was 1. Therefore, the total dietary antioxidant intake (TAC) was ranged between 0 (very poor) to 5 (high quality). Mediterranean dietary quality index (Med-DQI) and dietary phytochemical index were measured as described in our previous report [19]. Briefly, for calculating the Med-DQI daily intake of each of the seven food components including saturated fatty acids, cholesterol, olive oil, meats, fishes, cereals, vegetables and fruits assigned a score of 0, 1 or 2 and then final score was reported as a summation of all nutrient scores ranged between 0 and 14. The lower score of MED-DQI denoted a better nutrition quality and higher adherence to Mediterranean dietary pattern. For calculating the DPI, calories derived from high-phytochemical content foods including fruits, vegetables (except for potatoes), legumes, whole grains, nuts, seeds, fruit/vegetable juices, soy products are enumerated in this index. The higher score denoted the higher phytochemical content of diet.

### Statistics

SPSS software (statistical package for social analysis, version 18, SPSS Inc., Chicago, IL, USA) was used for analysis of data. The normality of data was tested by Kolmogorov-Smirnov test. The comparison of discrete and continuous variables between different quintiles of EDIP was performed by Chi- square test and analysis of variance (ANOVA) respectively. Odds ratios and 95% confidence intervals for the association between different quintiles of EDIP and biochemical parameters were estimated using logistic regression models, adjusting for confounders including age, BMI and presence of diabetes and myocardial infarction. All data are expressed as means ± SD or number and percent. *P* values less than 0.05 were considered as statistically significant.

## Results

Patients in higher quintiles of EDIP were younger and had higher BMI and prevalence of hyperlipidemia (P < 0.05, Table 1). The comparison of dietary indices among different quintiles of EDIP among patients showed that individuals in lower quintiles of EDIP had higher scores of vitamin E and total dietary antioxidant scores, higher dietary phytochemical score and lower dietary Mediterranean quality scores (Table 2). In comparison of OR and confidence interval (CI) for the association between EDIP and biochemical variables, in male subjects, highest scores of EDIP was associated with higher serum cholesterol, creatinine, BUN and Lp (a) concentrations (Table 3); moreover, being in third quintile of EDIP was associated with lower albumin concentrations compared with first quintile. In female subjects, lower HCT, higher creatinine and higher CRP concentrations were associated with higher EDIP scores (Table 4).

**Table 1:** General characteristics of candidate for CABG.

Quintiles of EDIP score						
Variable	1^st^ quintile	2^nd^ quintile	3^rd^ quintile	4^th^ quintile	5^th^ quintile	P value
	N = 90	N = 91	N = 92	N = 91	N = 90	
**Age (y)**	58.73± 8.48	62.24± 7.92	60.89± 8.77	58.58± 8.85	56.49± 9.52	**<0.001**
**Gender male [n (%)]**	16 (17.8)	30 (33)	20 (21.7)	29 (31.9)	24(26.7)	0.26
**BMI (kg/m**^**2**^**)**	26.69 ± 3.67	28.13± 4.24	26.76± 3.72	27.52± 4.43	28.08 ±3.68	**0.03**
**Diabetic [n (%)]**	55 (61.1)	56 (61.5)	51 (55.4)	57 (62.6)	43 (47.8)	0.12
**High education level [n (%)]**	14 16.1	10 (11.5)	12 (13.6)	13 (14.3)	17 (19.1)	0.74
**Smokers [n (%)]**	49 (54.4)	25 (27.5)	30 (32.6)	25 (27.5)	30 (33.7)	0.09
**Hyperlipidemia [n (%)]**	59 (65.5)	61 (67)	63 (68.5)	70 (76.9)	70 (77.8)	**0.023**
**Hypertension [n (%)]**	38 (42.2)	47 (51.6)	40 (43.5)	45 (49.5)	47 (52.8)	0.25
**MI [n (%)]**	46 (51.7)	48 (53.3)	46 (50)	50 (54.9)	43 (48.3)	0.62

BMI, body mass index; MI, myocardial Infarction; P value for discrete variables based on Chi-Square Test and for continuous variables based on ANOVA. Discrete and continuous variables data are presented as number (percent) and mean (SD). High educational attainment was defined as educational level more than 12 years.

**Table 2:** Comparison of the scores of DAQ, Mediterranean dietary quality index and dietary phytochemical index among different quintiles of EDIP.

		N	Mean	Std. Deviation	P
**Zn Score**	1^st^ quintile	90	0.96	0.18	0.43
	2^nd^ quintile	91	0.98	0.10	
	3^rd^ quintile	92	0.97	0.14	
	4^th^ quintile	91	0.98	0.10	
	5^th^ quintile	90	1.00	0.00	
**Vit E score**	1^st^ quintile	90	0.48	0.50	**0.04**
	2^nd^ quintile	91	0.34	0.47	
	3^rd^ quintile	92	0.34	0.47	
	4^th^ quintile	91	0.40	0.49	
	5^th^ quintile	90	0.40	0.50	
**Vit C score**	1^st^ quintile	90	0.98	0.10	0.23
	2^nd^ quintile	91	0.97	0.14	
	3^rd^ quintile	92	0.96	0.17	
	4^th^ quintile	91	1.00	0.00	
	5^th^ quintile	90	1.00	0.00	
**Vit A score**	1^st^ quintile	90	0.92	0.26	
	2^nd^ quintile	91	0.91	0.28	0.11
	3^rd^ quintile	92	0.89	0.31	
	4^th^ quintile	91	0.98	0.10	
	5^th^ quintile	90	0.93	0.25	
**Se Score**	1^st^ quintile	90	1.00	0.00	-
	2^nd^ quintile	91	1.00	0.00	
	3^rd^ quintile	92	1.00	0.00	
	4^th^ quintile	91	1.00	0.00	
	5^th^ quintile	90	1.00	0.00	
**DAQ score**	1^st^ quintile	90	4.40	0.69	**0.02**
	2^nd^ quintile	91	4.21	0.69	
	3^rd^ quintile	92	4.18	0.74	
	4^th^ quintile	91	4.30	0.51	
	5^th^ quintile	90	4.28	0.60	
**DPI**	1^st^ quintile	90	69.79	8.60	**<0.001**
	2^nd^ quintile	91	64.89	7.73	
	3^rd^ quintile	92	62.37	8.42	
	4^th^ quintile	91	61.80	9.52	
	5^th^ quintile	90	58.29	10.48	
**MEDQI**	1^st^ quintile	90	6.20	1.79	**0.004**
	2^nd^ quintile	91	6.32	1.81	
	3^rd^ quintile	92	6.54	1.80	
	4^th^ quintile	91	6.50	1.91	
	5^th^ quintile	90	6.57	1.93	

Zn, zinc; Se, selenium; DAQ, dietary antioxidant quality score; DPI, dietary phytochemical score; MEDQI, Mediterranean dietary quality score.

**Table 3:** Odd’s ratio (OR) and confidence interval (CI) for the association between EDIP and biochemical variables in male patients candidate for CABG.

Quintiles of EDII score					
Variable	1^st^ quintile	2^nd^ quintile	3^rd^ quintile	4^th^ quintile	5^th^ quintile
	N = 70	N = 53	N = 67	N = 61	N = 65
**HbA**_**1**_**C (%)**	1 (Ref.)	1.01(0.82–1.24)	0.92(0.74–1.14)	0.96 (0.78–1.18)	1.06 (0.88–1.27)
**TC (mg/dl)**	1 (Ref.)	1.02(0.98–1.05)	1.01(0.98–1.04)	1.01(0.98–1.03)	**1.04 (1.00–1.07)**
**TG (mg/dl)**	1 (Ref.)	0.99(0.98–1.00)	0.99(0.98–1.00)	0.99(0.99–1.00)	0.99(0.98–1.00)
**LDL (mg/dl)**	1 (Ref.)	0.98(0.95–1.01)	0.98(0.95–1.01)	0.98(0.96–1.01)	0.96(0.93–1.00)
**HDL (mg/dl)**	1 (Ref.)	0.99(0.93–1.06)	0.99(0.93–1.05)	1.01(0.96–1.07)	0.99(0.93–1.05)
**HCT (%)**	1 (Ref.)	1.06(0.98–1.15)	1.03(0.94–1.12)	1.02(0.94–1.12)	1.04(0.96–1.13)
**Albumin (g/dL)**	1 (Ref.)	0.49(0.15–1.61)	**0.33(0.10–1.04)**	0.63(0.20–1.92)	0.83(0.27–2.53)
**Creatinine (mg/dL)**	1 (Ref.)	3.84(0.46–31.98)	**6.78(0.92–49.66)**	2.97(0.37–23.26)	**8.41(1.54–25.60)**
**BUN (mg/dL)**	1 (Ref.)	**1.05(1.00–1.09)**	**1.05(1.01–1.09)**	**1.04(1.00–1.08)**	**1.03(0.99–1.07)**
**Lp (a) (mg/dL)**	1 (Ref.)	1.01(0.99–1.03)	**1.02(1.00–1.03)**	**1.01(0.99–1.03)**	1.01(0.99–1.02)
**CRP (mg/dL)**	1 (Ref.)	0.85(0.740.97)	0.84(0.75–0.95)	0.87(0.78–0.97)	0.88(0.79–0.98)
**Telomerase (U/l)**	1 (Ref.)	0.70 (0.37–1.34)	0.85 (0.47–1.04)	0.92 (0.5–1.72)	1.07 (0.59–1.96)
**Vitamin D (ng/ml)**	1 (Ref.)	1.03 (1.00–1.08)	1.03 (0.99–1.06)	1.03 (0.99–1.07)	1.05 (1.01–1.09)

Hb, hemoglobin; TC, total cholesterol; TG, triglyceride; LDL, low density lipoprotein cholesterol; HDL, high density lipoprotein cholesterol; HCT, hematocrit; BUN, blood urea nitrogen; CRP, C-reactive protein. The multivariate multinomial logistic regression was used for estimation of ORs and confidence interval (CI) with adjustment for the confounding effects of age, gender, BMI, educational attainment and presence of diabetes and myocardial infarction.* Indicates statistically significant values as P<0.05.

**Table 4:** Odd’s ratio (OR) and confidence interval (CI) for the association between EDII and biochemical variables in female patients candidate for CABG.

Quintiles of EDII score					
Variable	1^st^ quintile	2^nd^ quintile	3^rd^ quintile	4^th^ quintile	5^th^ quintile
	N = 16	N = 28	N = 20	N = 29	N = 24
**HbA**_**1**_**C (%)**	1 (Ref.)	1.05 (0.67–1.66)	1.21(0.71–2.04)	1.07(0.67–1.70)	0.93(0.57–1.53)
**TC (mg/dl)**	1 (Ref.)	1.00(0.98–1.02)	0.56(0.26–1.22)	0.77(0.35–1.70)	0.76(0.33–1.73)
**TG (mg/dl)**	1 (Ref.)	0.99(0.98–1.01)	1.11(0.95–1.30)	1.05(0.90–1.22)	1.05(0.89–1.24)
**LDL (mg/dl)**	1 (Ref.)	0.99(0.96–1.02)	1.77(0.82–3.81)	1.28(0.59–2.80)	1.32(0.58–3.01)
**HDL (mg/dl)**	1 (Ref.)	1.05(0.96–1.14)	1.80(0.83–3.89)	1.36(0.62–2.99)	1.38(0.60–3.16)
**HCT (%)**	1 (Ref.)	1.06(0.85–1.31)	**0.74 (0.51–6.97)**	0.95(0.75–1.20)	0.88(0.68–1.13)
**Albumin (g/dL)**	1 (Ref.)	1.27(0.14–9.64)	1.53(0.11–20.02)	0.74(0.08–6.85)	0.30(0.02–3.14)
**Creatinine (mg/dL)**	1 (Ref.)	7.22(0.23–9.10)	3.74(0.25–9.2)	**8.84 (0.59–12.91)**	**7.42(0.46–11.46)**
**BUN (mg/dL)**	1 (Ref.)	0.96(0.90–1.04)	0.92(0.85–1.01)	0.96 (0.89–1.03)	0.91(0.84–0.99)
**Lp (a) (mg/dL)**	1 (Ref.)	1.05(0.98–1.02)	0.99(0.96–1.01)	0.99 (0.97–1.02)	0.99(0.97–1.02)
**CRP (mg/dL)**	1 (Ref.)	0.81(0.55–1.21)	**1.24 (0.97–1.59)**	1.09 (0.85–1.39)	1.12(0.87–1.46)
**Telomerase (U/l)**	1 (Ref.)	2.62 (0.63–10.86)	**4.16 (0.97–17.84)**	2.56 (0.6–10.83)	0.97 (0.19–4.81)
**Vitamin D (ng/ml)**	1 (Ref.)	0.99 (0.94–1.05)	1.00 (0.94–1.06)	0.99 (0.94–1.05)	0.99 (0.94–1.05)

Hb, hemoglobin; TC, total cholesterol; TG, triglyceride; LDL, low density lipoprotein cholesterol; HDL, high density lipoprotein cholesterol; HCT, hematocrit; BUN, blood urea nitrogen; CRP, C-reactive protein. The multivariate multinomial logistic regression was used for estimation of ORs and confidence interval (CI) with adjustment for the confounding effects of age, gender, BMI, educational attainment and presence of diabetes and myocardial infarction.* Indicates statistically significant values as P < 0.05.

## Discussion

In the current work, high empirically developed inflammatory potential of diet was associated with higher body mass index, higher prevalence of hyperlipidemia, lower score of vitamin E and antioxidant score of diet and lower DPI and Med-DQI in patients candidate for CABG. Moreover, among biochemical variables, highest scores of EDIP was associated with higher serum cholesterol, creatinine, BUN and Lp (a) and lower albumin concentrations in men and lower HCT, higher creatinine and higher CRP concentrations in women. This is the first study reveals the association between EDIP of diet and numerous metabolic risk factors of CVD and also dietary indices among patients candidate for CABG.

EDIP of diet, give a perspective of the potential of diet in inducing inflammation mostly based on increased interleukin-6 (IL-6), C-reactive protein (CRP), and tumor necrosis factor a receptor 2 (TNFaR2) concentrations [10]. Obesity is a well- known low-grade chronic inflammatory condition mostly because of adipose tissue derived cytokines development and accumulation in the body [20]. The association between pro-inflammatory potential of diet and incidence of overweight and obesity has been studied and revealed before; in the study by Ramallal R among adults of SUN cohort, being in highest pro-inflammatory diet index make people 1.32 times more likely to being obese annually compared with lowest quartile (anti-inflammatory diet) [21]. Another population based report among 7236 participants, dietary inflammatory index was directly associated with the incidence of general and central obesity even after adjustment for the known risk factors [22]. The association between obesity and inflammation can be bidirectional meaning that adiposity can induce inflammation, while a pro-inflammatory diet can induce adiposity and therefore this will make a vicious cycle between obesity and inflammation [23]; accordingly, in our previous work, we reported the higher DII in association of serum lipids and CRP concentrations among candidates of CABG [24]. Inflammation leads to HDL reduction and LDL and VLDL production in the cells via impairment in the reverse cholesterol transport system [25]; another possible mechanism is triggering receptor expressed on myeloid cells-1 (TREM-1) induced dyslipidemia and consequently, fat deposition and pro-inflammatory cytokines production [26].

In the current work, EDIP of diet was negatively associated with vitamin E and total dietary antioxidant scores and also was in negative association with DPI and Med-DQI in patients. These findings showed that EDIP is a precise measure of other dietary indices as expected; same as our results, Hodje et al [27] reported an inverse association between Mediterranean dietary score and DII while higher Mediterranean dietary score denoted more healthy diet. In another work by Bawaked et al [28], higher DII of diet was associated with lower adherence to Mediterranean diet, lower antioxidant capacity of diet and high energy density in youth. These findings confirm that EDIP of diet has a precise association with other dietary indices and is a reliable indicator of unhealthy diet.

Lower serum albumin in third quintile of EDIP compared with first quintile in women is another finding indicting the association between diet and inflammation. Albumin is a negative acute phase proteins and inflammation is a suppressor of its synthesis [29]. It has been shown that anti-inflammatory and antioxidant nutritional interventions are able to increase serum albumin concentrations and to reduced hypo-albuminemia [30]. Higher serum BUN and creatinine were associated with higher inflammatory potential of diet in patients candidate for CABG. We also reported similar results in our previous work indicating positive association between DII and serum creatinine [24]. Several other studies have also reported consistent results about the potential of inflammatory diet in inducing chronic kidney disease and reduced kidney function by increasing CRP concentrations, reducing glomerular filtration rate (GFR) and increase in BUN and creatinine [31, 32].

We should also address several limitations of the current study; first of all, the self-reported dietary information obtained by food frequency questionnaire could have a potential recall bias. However, the questionnaire has been validated previously. Secondly, we did not measure WHR or WC as indicators of central adiposity. Taking together, the study’s relatively large sample size and inclusion of multiple confounders in the statistical model are potent strengths of the current study. Moreover, this is the first study evaluated the association between EDIP and CVD risk factors in patients candidate for CABG.

## Conclusion

**Fig 1:**
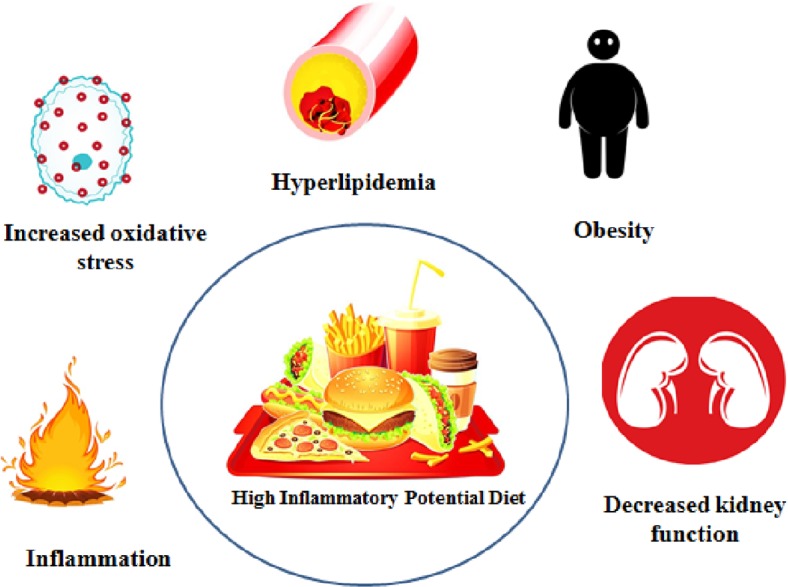
The summarized graphical abstract of the metabolic disorders associated with high inflammatory potential of diet.

We reported that EDIP is a potent predictor of obesity, dyslipidemia and cardio-metabolic risk factors among patients candidate for CABG. The summarized conceptual findings of the work have been presented in Fig 1. Additionally, EDIP of diet was in inverse association with two dietary indices including dietary anti-oxidant quality score and dietary phytochemical index. Taking together, EDIP could be assumed as a precise nutritional tool for estimating the CVD related risk factors among patients candidate for CABG.

### Ethical approval and consent to participate

All participants signed a written informed consent approved by the Institutional Review Board of Tehran University of Medical Sciences. The study design and protocol was approved by the ethical committee of Tehran and Tabriz University of Medical Sciences.

## Supporting information

S1 Dataset (SAV)

## Abbreviations

ANOVA: analysis of variance
CAD: coronary artery disease
CVD: cardiovascular disease
DAQs: dietary antioxidant quality score
DPI: dietary phytochemical index
EDIP: empirically developed dietary inflammatory potential
HDL: high density lipoprotein cholesterol
hs-CRP: high sensitive C reactive protein
LDL: low density lipoprotein cholesterol
Lp: lipoprotein
Med-DQI: Mediterranean dietary quality index
TC: total cholesterol
